# Efficacy of the cardiac glycoside digoxin as an adjunct to csDMARDs in rheumatoid arthritis patients: a randomized, double-blind, placebo-controlled trial

**DOI:** 10.3389/fphar.2024.1445708

**Published:** 2024-10-21

**Authors:** Nageh A. El-Mahdy, Mariam G. Tadros, Thanaa A. El-Masry, Ammena Y. Binsaleh, Nawal Alsubaie, Amani Alrossies, Medhat I. Abd Elhamid, Enas Y. Osman, Hadeel M. Shalaby, Dalia S. Saif

**Affiliations:** ^1^ Department of Pharmacology and Toxicology, Faculty of Pharmacy, Tanta University, Tanta, Egypt; ^2^ Department of Pharmacy Practice, Faculty of Pharmacy, Sinai University - Arish Branch, Arish, Egypt; ^3^ Department of Pharmacy Practice, College of Pharmacy, Princess Nourah bint Abdulrahman University, Riyadh, Saudi Arabia; ^4^ Department of Pharmacology, Faculty of Medicine, Al-Azhar University, Cairo, Egypt; ^5^ Department of Clinical Pathology, Faculty of Medicine, Menoufia University, Menoufia, Egypt; ^6^ Department of Rheumatology, Rehabilitation, and Physical Medicine, Faculty of Medicine, Menoufia University, Menoufia, Egypt

**Keywords:** rheumatoid arthritis, digoxin, inflammatory markers, angiogenesis, ACR

## Abstract

**Background:**

Inflammation and angiogenesis are two main mechanisms that act as mutual pathways in rheumatoid arthritis (RA). This work aimed to study the efficacy of digoxin as an adjunct therapy to conventional synthetic disease-modifying anti-rheumatic drugs (csDMARDs) in active RA patients.

**Methods:**

In a randomized, double-blinded, placebo-controlled study, 60 adult patients with active RA received a placebo or digoxin (0.25 mg every other day) combined with csDMARDs for 6 months. The American College of Rheumatology (ACR) 20, ACR50, and ACR70 response rates and the disease activity score (DAS28) were assessed for patients. Flow cytometric analysis of Th17 cells and serum concentrations of IL-17A, IL-23, HIF-1α, and VEGF were evaluated before and after three and 6 months of therapy.

**Results:**

Following three and 6 months of digoxin therapy combined with csDMARDs, significant differences were detected in laboratory and clinical parameters relative to the control group. After 6 months, 83.3% of patients in the digoxin group, compared to 56.7% in the control group, achieved an ACR20 response (*p* = 0.024). The digoxin group had a significantly higher percentage of patients who achieved DAS28 remission after 6 months (*p* = 0.024). Notable improvements in the Health Assessment Questionnaire Disability Index, ACR50, and ACR70 were detected in the digoxin group.

**Conclusion:**

Digoxin was well tolerated and exerted profound immunomodulatory and anti-inflammatory effects in RA patients, and may also exhibit anti-angiogenic properties, indicating that it might be an effective adjunct to csDMARDs in treating RA.

**Clinical Trial Registration:**

clinicaltrials.gov, identifier NCT04834557.

## 1 Introduction

Rheumatoid arthritis (RA) is a chronic, debilitating, progressive, autoimmune disease affecting synovial joints, inducing synovial inflammation, leukocyte infiltration, excessive release of various inflammatory mediators and autoantibodies, pannus formation, bone erosion, and cartilage destruction, with resultant joint deformity (Guo and Chen, 2020; Schinocca et al., 2021; Scott et al., 2010).

Many lines of evidence have shown that inflammation and angiogenesis are the two main mechanisms of RA, which act as mutual pathways rather than individual processes (Elshabrawy et al., 2015; Guo et al., 2018).

T helper 17 (Th17) cells have potential and direct involvement in RA’s pathogenesis. Th17 cells selectively secrete interleukin-17A (IL-17A), which triggers cytokine mediators of the inflammatory response, contributing to a persistent inflammatory response and subsequent tissue destruction (Alunno et al., 2015).

Th17 cell differentiation and activation are induced by the inflammatory cytokine mediator IL-23, with subsequent IL-17A production, which is preferentially implicated in RA’s pathogenesis. Therefore, the association between IL-23/IL-17A signaling and inflammatory arthritis development represents a crucial immunological pathway and a promising target for therapeutic intervention in various arthritic conditions (Schinocca et al., 2021).

Vascular endothelial growth factor (VEGF) stands out as the first identified vasculogenic factor, which induces new blood vessel formation through migration and proliferation of endothelial cells (Elshabrawy et al., 2015; Zisman et al., 2021). VEGF expression is amplified by hypoxia-inducible factor-1α (HIF-1α), a transcription factor that controls cell responses to low oxygen levels (George et al., 2020; Gong et al., 2019; Saponaro et al., 2013). Therefore, HIF-1α and VEGF are potential therapeutic targets for angiogenesis in RA.

Peripheral blood biomarkers, including neutrophil to lymphocyte ratio (NLR), platelet to lymphocyte ratio (PLR), lymphocyte to monocyte ratio (LMR), systemic immune inflammation index (SII), and systemic inflammation response index (SIRI), are promising biomarkers for immune activation and inflammation that are strongly associated with the disease activity of several rheumatic diseases (Hao et al., 2017; Kumarasamy et al., 2019; Li et al., 2021; Mirestean et al., 2023; Sejópoles et al., 2023; Su et al., 2021; Targonska-Stepniak and Grzechnik, 2023; Urbanowicz et al., 2021; Zhou et al., 2023).

The effectiveness of conventional synthetic (cs) DMARDs (disease-modifying anti-rheumatic drugs) has acquired considerable attention as they can attenuate disease activity and delay joint deformation. However, even now, many patients either do not respond or respond only partially to these compounds, and complete disease remission in the long term is not achieved for many patients, which requires the development of new therapeutic options (Grennan et al., 2001; Guo et al., 2018).

Digoxin is a cardiovascular glycoside used for the management of heart failure and other cardiac complications (Jordaens et al., 1997). Its usage in the cardiac area has accounted for the majority of our knowledge; however, recent studies showed that digoxin has beneficial clinical therapeutic utilization in non-cardiac conditions, including viral infection, cancer, steatohepatitis, and autoimmune diseases (Jamshed et al., 2023). Intriguingly, reports propose that digoxin reduced the severity of arthritis and autoimmune encephalomyelitis in the experimental model and suppressed Th17 differentiation by inhibiting the retinoic acid-related orphan receptor γt (RORγt) transcriptional activity (Peters et al., 2011; Seki and Nishizawa, 2016). In this regard, digoxin-mediated inhibition of RORγt may be valuable in treating RA (Saeed et al., 2020).

Based on the aforementioned observations, we proposed to investigate the immunomodulatory, anti-inflammatory, and anti-angiogenic properties of digoxin when co-administered with csDMARDs in patients with RA who exhibit moderate to high disease activity with no other problems and its association with therapeutic response and improvement of disease activity to provide worthy insights for RA treatment.

## 2 Methods

### 2.1 Study design and setting

This study was a prospective, randomized, double-blinded, placebo-controlled clinical trial performed on Egyptian RA patients. The Institutional Review Board of the Faculty of Medicine, Menoufia University, approved the study protocol under the IRB# PMRR2A. The study adhered to the ethical basics and procedures established in the 1964 Declaration of Helsinki and any later changes. Eligible participants were comprehensively informed about the study protocol before participation and were required to provide written confirmation of their informed consent that followed the guidelines declared by the local ethics committee. The clinical study was conducted at the Physical Medicine, Rheumatology, and Rehabilitation Department from November 2021 to September 2022 and was recorded at “ClinicalTrials.gov” with a unique identifier number of NCT04834557. The present article focused on analyzing and examining data, and reporting results from only two of the three intervention groups enrolled in this clinical trial.

### 2.2 Study participants

**FIGURE 1:**
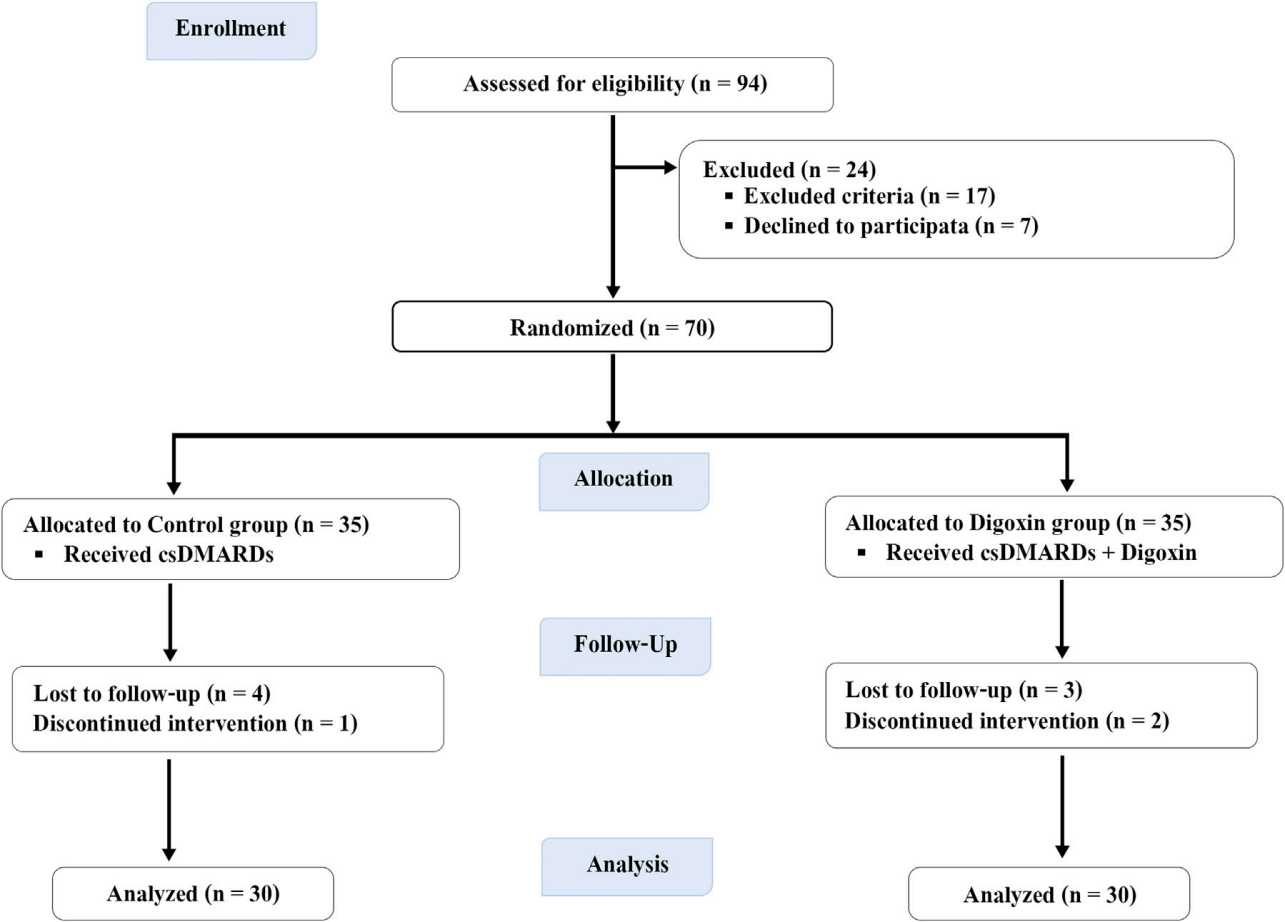
Flow diagram of study participants throughout the study.

Seventy patients with a clinically established diagnosis of RA fulfilling the 2010 American College of Rheumatology/European League Against Rheumatism (ACR/EULAR) classification criteria were recruited (Aletaha et al., 2010) (Figure 1).

RA disease activity status was identified by measuring the disease activity score-28 based on the levels of C-reactive protein (CRP) (DAS28-CRP) > 3.2. Patients who exhibited moderate to high disease activity and were treated with csDMARDs were eligible to participate in the study.

Exclusion criteria included; hypersensitivity to digoxin, other autoimmune diseases, patients on biologic DMARDs or refusing to give informed consent, cases of impaired liver functions and kidney functions, oral prednisolone >10 mg/day, pregnancy and lactation, as well as the existence of other comorbidities, including malignancies, any cardiovascular, psychiatric or neurological diseases, diabetes mellitus, active infections, and other inflammatory diseases.

### 2.3 Demographic data

Demographic data were reported for all RA patients, including age, gender, smoking, height, weight, body mass index (BMI), disease duration, and medications received (Table 1).

**TABLE 1:** Baseline demographic, clinical, and laboratory parameters of the study population.

Characteristics	Control group (n = 30)	Digoxin group (n = 30)	95% CI	*p*-value
Demographic Characteristics				
Age (years)	51.1 ± 9.25	51.3 ± 9.39	−4.9, 4.7	0.956^a^
Gender, n (%) Male Female	6 (20%) 24 (80%)	7 (23.3%) 23 (76.7%)	0.17, 3.66	0.754^c^
Smoking	6 (20%)	5 (16.7%)	−0.11, 2.17	0.739^c^
Weight (Kg)	71.5 (68–73.7)	72 (68–77.3)	0.78, 0.95	0.812^b^
Height (cm)	170.9 ± 4.93	171.1 ± 5.69	−3, 2.5	0.856^a^
BMI (kg/m^2)	24.6 (24.2–25.1)	24.8 (24.5–25)	0.23, 0.47	0.394^b^
Clinical Characteristics				
Disease duration (years)	9.43 ± 3.58	9.43 ± 3.51	−1.8, 1.8	1.00^a^
Tender joint count (TJC, 28 count)	11 (7.8–13)	11 (8–13.3)	0.68, 0.89	0.784^b^
Swollen joint count (SJC, 28 count)	7.23 ± 3.07	7.50 ± 3.16	−1.9, 1.3	0.741^a^
Patient’s global assessment of disease activity (0–100 mm, VAS)	53.5 ± 11.5	53.9 ± 11.6	−6.3, 5.6	0.911^a^
Physician’s global assessment of disease activity (0–100 mm, VAS)	45.1 ± 11.3	45.2 ± 11.5	−6, 5.8	0.973^a^
Patient’s assessment of pain (0–100 mm, VAS)	51.7 ± 11.6	51.9 ± 11.7	−6.3, 5.7	0.930^a^
DAS28-CRP score	5.40 ± 0.83	5.46 ± 0.78	−0.47, 0.36	0.779^a^
DAS28 class, n (%) Moderate disease activity (>3.2–5.1), n (%)	10 (33.3%)	11 (36.7%)	0.06, 2.51	0.787^c^
High disease activity (>5.1), n (%)	20 (66.7%)	19 (63.3%)	0.06, 2.51	0.787^c^
DAS28-ESR score	5.94 ± 0.88	6.01 ± 0.80	−0.5, 0.37	0.763^a^
Morning stiffness (min)	66.5 (61.8–74)	66.5 (62–71.5)	0.92, 1.0	0.970^b^
HAQ-DI score	1.88 ± 0.49	1.89 ± 0.43	−0.26, 0.23	0.899^a^
Systolic Blood pressure (mmHg)	124 (122–125)	123 (122–125.5)	0.82, 0.98	0.887^b^
Diastolic Blood pressure (mmHg)	74 (72–74)	73.5 (72.8–74)	0.95, 1.0	0.964^b^
RA-associated serum markers				
ESR (mm/hr)	45.3 ± 14.2	46.9 ± 11.0	−8.1, 5.1	0.643^a^
CRP (mg/L)	24 (16.8–31.3)	24.5 (17.8–34.5)	0.60, 0.83	0.739^b^
RF-positive, n (%)	23 (76.7%)	24 (80%)	0.12, 1.18	0.754^c^
ACPAs-positive, n (%)	17 (56.7%)	21 (70%)	−1.08, 2.14	0.284^c^
Other Laboratory parameters				
Total cholesterol (mg/dL)	167 (160.8–173.7)	166.9 (162.6–169.7)	0.60, 0.83	0.756^b^
HDL (mg/dL)	45 (39.8–47.3)	43 (41.8–45)	0.01, 0.32	0.117^b^
LDL (mg/dL)	96.5 (92–105.5)	97 (95–100.3)	0.37, 0.63	0.538^b^
TGs (mg/dL)	126 (115.8–140.5)	132.5 (126.8–137)	0.20, 0.43	0.386^b^
Serum albumin (g/dL)	3.6 (3.5–3.8)	3.6 (3.5–3.8)	0.23, 0.47	0.375^b^
AST (IU/L)	23 (20–32)	24.5 (22–29)	0.29, 0.54	0.419^b^
ALT (IU/L)	25.2 ± 9.30	26.47 ± 6.41	−5.4, 2.8	0.531^a^
BUN (mg/dL)	13 (11–16)	14 (11–15)	0.60, 0.83	0.721^b^
Serum creatinine (mg/dL)	0.8 (0.7–0.97)	0.8 (0.62–0.91)	0.17, 0.40	0.217^b^
Methotrexate, n (%)	30 (100%)	30 (100%)	—	—
Hydroxychloroquine, n (%)	18 (60%)	17 (56.7%)	0.19, 0.95	0.793^c^
Leflunomide, n (%)	18 (60%)	20 (66.7%)	0.22, 0.89	0.592^c^
Oral GC, Prednisolone, n (%)	16 (53.3%)	15 (50%)	0.16, 1.11	0.796^c^

Data are presented in terms of mean ± SD, median (inter-quartile range IQR), or number and percentages.

^a^ Unpaired student’s *t*-test.

^b^ Mann-Whitney U test.

^c^ Chi-square test.

Abbreviations: RA, rheumatoid arthritis; BMI, body mass index; VAS, visual analogue scale; DAS28-ESR, Disease Activity Score-28-erythrocyte sedimentation rate; DAS28-CRP, Disease Activity Score-28-C-reactive Protein; HAQ DI, health assessment questionnaire disability index; ESR, erythrocyte sedimentation rate; CRP, C-reactive Protein; RF, rheumatoid factor; ACPAs, anti-citrullinated peptide antibodies; HDL, High-density lipoprotein; LDL, Low-density lipoprotein; TGs, Triglycerides; AST, aspartate aminotransferase; ALT, alanine aminotransferase; IU, international units; BUN, blood urea nitrogen; GCs, Glucocorticoids; n, number; 95%CI, 95% Confidence Interval.

### 2.4 Clinical assessment and follow-up

Each patient underwent a comprehensive clinical and thorough assessment by a rheumatologist and was given a blood sample at baseline, three and 6 months after treatment. Medical history was reported to assert that there was no interfering or interacting diseases or drugs. Comprehensive clinical evaluation of disease activity was done at the beginning of the study and after three and 6 months of treatment, including tender and swollen 28-joint counts (TJC28 and SJC28), joint movement assessment, the patient’s as well as physician’s global assessment (PtGA, PhGA; visual analog score from 0 to 100 mm), patient’s assessment of pain (0–100 mm, VAS), disease activity status assessed using DAS28-CRP and DAS28-ESR, morning stiffness, and the quality of life (QOL) of the patients was assessed using the Health Assessment Questionnaire Disability Index (HAQ-DI) and given a score from 0 (without any difficulty) to 3 (unable to do) where higher scores indicate poor QOL (Fries et al., 1980). Blood pressure was also assessed, with both systolic and diastolic values (mmHg) for all patients (Table 1).

Laboratory investigations were also performed, including measurements of CRP (mg/dL), ESR (mm/h), rheumatoid factor (RF), anti-citrullinated peptide antibodies (ACPAs), complete blood count (CBC), and differential. In addition, lipid profile, liver, and renal function tests were performed to monitor drug tolerability and toxicity (Table 1).

### 2.5 Sample size

Based on the study reported by Saeed et al. (2020), the sample size was calculated on the assumption that a two-sided significance is 0.05 and a power of 90% is used with a 95% confidence interval; therefore, 30 participants were required for each treatment group.

### 2.6 Randomization and blinding

A simple randomization process was employed according to the Consolidated Standards of Reporting Trials (CONSORT) guidelines to shuffle the patients who fulfilled the inclusion criteria into either the control group or the digoxin group based on a 1:1 ratio. Patients, the physician who referred them, the data collectors, and the statistician were blinded to avoid bias in the study. During the research, if the patient’s study drug caused any adverse effect requiring immediate emergency care, the rheumatologist involved was not blinded, and after breaking the blinding, the patient was excluded from the study.

### 2.7 Therapeutic intervention

Thirty participants in the active control group were treated with csDMARDs, including methotrexate 7.5–15 mg/week with or without hydroxychloroquine 400 mg/day or leflunomide 10 mg/day plus one placebo tablet every other day for 6 months, whereas the other 30 participants in the digoxin group were treated with csDMARDs, including methotrexate 7.5–15 mg/week with or without hydroxychloroquine 400 mg/day or leflunomide 10 mg/day plus digoxin 0.25 mg every other day for 6 months. Oral glucocorticoids such as prednisolone (≤10 mg/day) were allowed. The csDMARDs were initiated prior to study enrollment and maintained at a stable dose throughout the six-month trial for both the active control and digoxin groups. The medication refill rate was used to assess the patient’s compliance with the prescribed treatment regimen, and non-adherent participants were excluded from the study.

### 2.8 Outcome assessment

The primary efficacy outcome focused on a clinically significant improvement in ACR20 response rates which correspond to a minimum of 20% improvement in tender and swollen 28-joint counts (TJC28 and SJC28), and at least three of the five parameters: HAQ-DI, patient’s and physician’s global assessment of arthritis, pain assessment, and CRP or ESR levels (Felson et al., 1995), with complementary evaluation of disease activity using the clinical disease activity index (CDAI) (Greenberg et al., 2009) and changes in acute phase reactants CRP and ESR levels that indicate inflammatory activity, and their reduction alongside improvement in ACR20 strengthens the evidence for the treatment’s effectiveness in reducing inflammation after three and 6 months of treatment. The CDAI, calculated by summing tender and swollen 28-joint counts, as well as patient’s and physician’s global assessments, offers a comprehensive, readily applicable assessment for evaluating disease activity and treatment response in RA patients with scores defined as: remission (≤2.8), low disease activity (>2.8 to ≤10.0), moderate disease activity (>10.0 to ≤22.0), and high disease activity (>22.0) (Aletaha and Smolen, 2005). The secondary efficacy outcomes, evaluated at the same time points, were defined as the assessed improvement in disease activity, which included ACR50 and ACR70 response rates, which correspond to a minimum of 50% and 70% improvement, respectively, in tender and swollen 28-joint counts, and in three of the following: HAQ-DI, patient’s and physician’s global assessment of arthritis, pain assessment, and CRP or ESR levels (Felson et al., 1995). Also, the study considered the EULAR response criteria that depend on the DAS28-CRP disease activity index and the change that occurred in it through the treatment period (Verhoeven et al., 2000). The other assessment indicators include DAS28-ESR, morning stiffness, simplified disease activity index (SDAI) (Smolen et al., 2003), and hematologic parameters calculated from total and differential complete blood counts (CBC), including NLR, PLR, LMR, SII, and SIRI, which are correlated with disease activity and prognosis.

Other outcomes involved the quantification of IL-17A, IL-23, HIF-1α, and VEGF serum levels together with the proportions of Th17 cells (IL-17A+ CD4+ T cells) at the beginning of the study and after the treatment period to investigate the potential biological changes induced by the trial medications.

Safety outcome measures were primarily evaluated throughout the study by recording the experienced adverse effects and their severity (mild, moderate, or severe) in both the control and digoxin groups. The patient’s medical history was carefully assessed, and they were comprehensively educated about the potential adverse effects of digoxin and were required to note any adverse events they encountered throughout the treatment course. In addition, clinical and physical examinations, as well as electrocardiograms (ECG), were carried out.

### 2.9 Sample collection and measurements

Venous samples were obtained from RA patients before as well as three and 6 months post-treatment initiation, and the samples were categorized into two parts: the whole blood portion, which was used to analyze Th17 cells by flow cytometry, and the serum portion, which was used for ELISA analysis.

The serum levels of cytokines IL-17A (Sun Red International trade company, Shanghai, China; 201-12–0048) and IL-23 (Cloud-Clone Corp Co., United States; SEA384Hu), as well as the angiogenic factors HIF-1α (Sun Red International trade company, Shanghai, China; 201-12–0423) and VEGF (Cloud-Clone Corp Co., United States; SEA143Hu), were measured by specific human enzyme-linked immunosorbent assay (ELISA) kits based on the manufacturer’s instructions utilizing a Biotek ELx800 UV-Vis microplate reader. The values were presented in picograms per milliliter (pg/mL) with standard deviation (SD).

### 2.10 Flow cytometric analysis of Th17 cells

**FIGURE 2:**
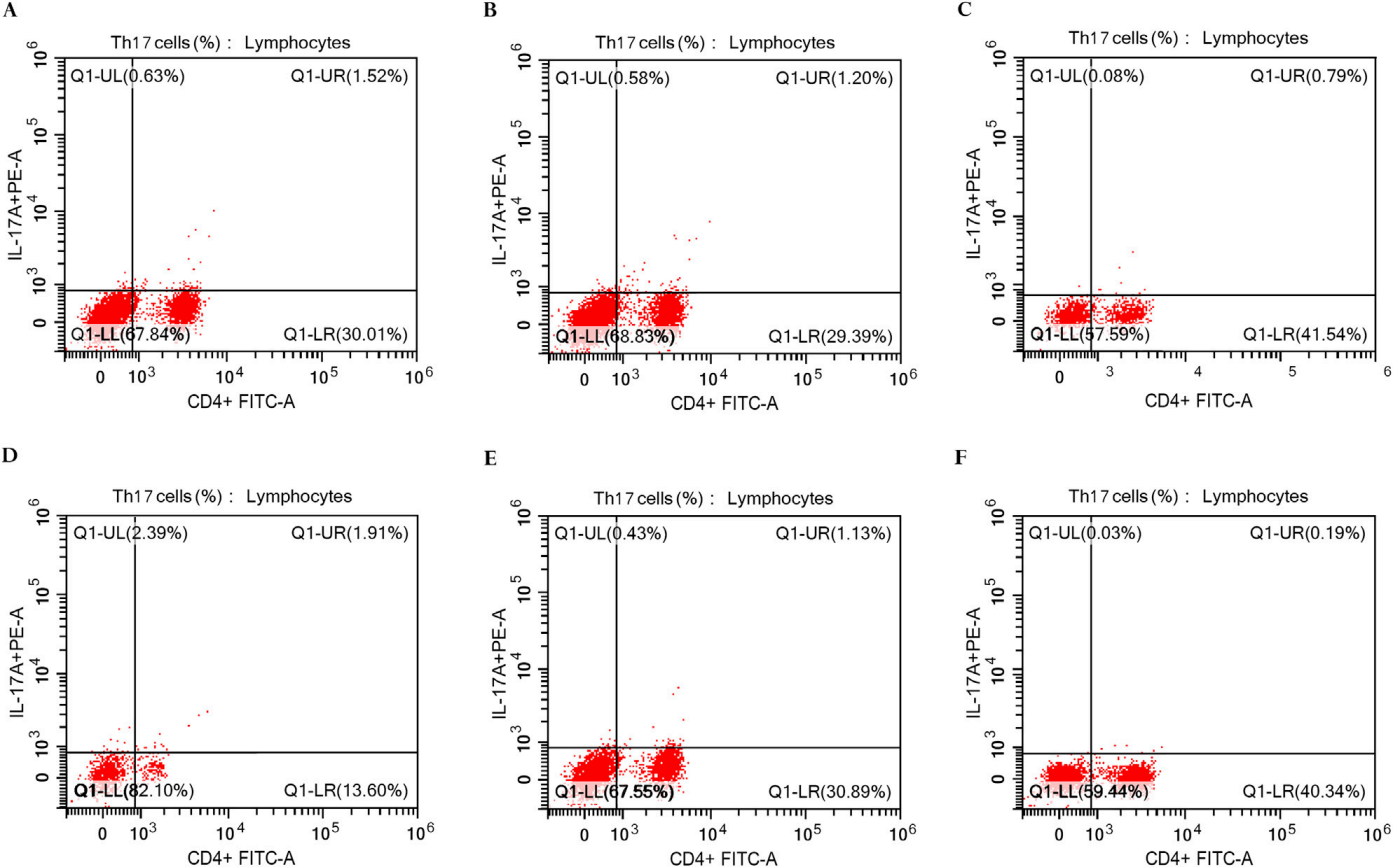
Flow cytometric analysis of Th17 cells in PBMCs of rheumatoid arthritis patients. **(A)** The percentage of IL-17A+ CD4^+^ T cells in RA patients (1.52%) at baseline in the control group (n = 30). **(B)** The percentage of IL-17A+ CD4^+^ T cells in RA patients (1.20%) after 3 months of treatment in the control group (n = 30). **(C)** The percentage of IL-17A+ CD4^+^ T cells in RA patients (0.79%) after 6 months of treatment in the control group (n = 30). **(D)** The percentage of IL-17A+ CD4^+^ T cells in RA patients (1.91%) at baseline in the digoxin group (n = 30). **(E)** The percentage of IL-17A+ CD4^+^ T cells in RA patients (1.13%) after 3 months of treatment in the digoxin group (n = 30). **(F)** The percentage of IL-17A+ CD4^+^ T cells in RA patients (0.19%) after 6 months of treatment in the digoxin group (n = 30).

The peripheral blood mononuclear cells (PBMCs) were isolated using Ficoll-Hypaque density gradient centrifugation (GE Healthcare Bio-Sciences, Uppsala, Sweden), and then the cells were washed in phosphate-buffered saline (PBS). Following staining with FITC-conjugated anti-CD4 (Beckman Coulter Life Sciences, Indianapolis, United States; IM0448U), fixation and permeabilization of cells were done using the Fixation/IntraPrep Permeabilization Kit (Beckman Coulter Company, France; A07803) and then stained intracellularly with PE-conjugated anti-human IL-17A (BD Biosciences Pharmingen, United States; 560436) according to the manufacturer’s protocol. Cell analysis was conducted utilizing the CYTOFLEX Flow Cytometer (Beckman Coulter Life Sciences, Indianapolis, United States) and CytExpert Software version 2.5 (Figure 2).

### 2.11 Statistical analysis

Statistical analyses were conducted using IBM SPSS Statistics version 27.0 software (IBM Corporation, Armonk, New York, United States). Quantitative data were tested for normality using the Shapiro-Wilk test. Quantitative data were exhibited as means and standard deviations (SD) or medians and inter-quartile range (IQR), while qualitative data were displayed as frequencies (n) and percentages (%). Paired and unpaired Student's *t*-tests were employed for quantitative comparisons of parametric data, while Mann-Whitney U and Wilcoxon signed-rank tests were employed for quantitative comparisons of nonparametric data. Chi-Square (χ2) and Fisher’s exact tests were utilized for analyzing qualitative variables. Pearson’s correlation coefficient (r) for parametric data and Spearman’s rank correlation coefficient (r) for nonparametric data, evaluated the relationships between measured variables. Multivariate logistic and linear regression models were used to describe the associations of predictors (baseline demographic characteristics) with the outcomes. The results were expressed as odds ratios (ORs) in logistic regression models and as regression coefficients in linear regression models. The 95% confidence interval (95% CI) was calculated, and the statistical significance was determined at *p* < 0.05.

## 3 Results

### 3.1 Baseline evaluation

Out of the 94 encountered patients, 24 subjects were excluded: 17 patients due to disease comorbidities including cardiac diseases, endocrinal disorders, and impaired renal function (n = 17); 7 patients declined participation in the study (n = 7); therefore, the remaining 70 patients were enrolled and randomized to both groups in the study. Ten participants (5 in each group) were dropped out and their initial data were omitted from the final analysis which included 30 participants in each group. Only participants who adhered to the study protocol throughout the trial were included, while data from participants who deviated from the protocol were excluded from the analysis.

Baseline assessment showed that the two study groups displayed no significant differences concerning demographic features, clinical and laboratory variables, or csDMARDs received in each group (*p* > 0.05), as presented in Table 1.

### 3.2 Clinical efficacy outcome

After 3 months of treatment, digoxin administration led to a marked increase in the ACR20 response relative to the control group (66.7% vs. 36.7%, *p* = 0.020). Consistently, improved efficacy was observed at 6 months of treatment, with significantly higher rates of ACR50 (46.7% vs. 20%, *p* = 0.028) and ACR70 (26.7% vs. 6.7%, *p* = 0.038) responses in the digoxin group patients relative to patients in the control group (Table 2). Table 2 also demonstrates that after three and 6 months of treatment, patients in the digoxin group exhibited a statistically significant enhancement in their EULAR response criteria in contrast to the control group (*p* < 0.001 and *p* = 0.008, respectively).

**TABLE 2:** Clinical response in rheumatoid arthritis patients after three and 6 months of treatment.

Clinical characteristics	Control group (n = 30)			Digoxin group (n = 30)			*p*-value**			
	3 months	6 months	*p*-value*	3 months	6 months	*p*-value*	A vs. C	95%CI	B vs. D	95%CI
Median percentage change in DAS28-CRP from baseline	−6.36 (−11.6–−4.3)	−14.9 (−25.3–−10.6)	**<0.001** ^a^	−18.3 (−27.8–−15.6)	−40.2 (−52.5–−26.9)	**<0.001** ^a^	**<0.001** ^b^	8.7, 16.3	**<0.001** ^b^	13.3, 27.5
ACR20, n (%)	11 (36.7%)	17 (56.7%)	0.196^c^	20 (66.7%)	25 (83.3%)	0.233^c^	**0.020** ^c^	0.001, 0.96	**0.024** ^c^	0.005, 0.94
ACR50, n (%)	3 (10%)	6 (20%)	0.470^c^	10 (33.3%)	14 (46.7%)	0.429^c^	**0.028** ^c^	0.009, 0.76	**0.028** ^c^	0.01, 0.96
ACR70, n (%)	0	2 (6.7%)	0.472^c^	0	8 (26.7%)	**0.008** ^c^	—	—	**0.038** ^c^	0.02, 0.66
EULAR response, n (%) Good response Moderate response No response	0 5 (16.7%) 25 (83.3%)	6 (20%) 10 (33.3%) 14 (46.7%)	**0.005** ^c^	6 (20%) 17 (56.7%) 7 (23.3%)	17 (56.7%) 8 (26.7%) 5 (16.7%)	**0.012** ^c^	**<0.001** ^c^	0.0, 0.049	**0.008** ^c^	0.002, 0.19

Data are presented in terms of median (inter-quartile range IQR) or number and percentages.

^a^ Wilcoxon signed-rank test.

^b^ Mann-Whitney U test.

^c^ Chi-square test.

*Within group comparison (3 months vs. 6 months).

**Between groups comparison where A vs. C represent comparison between groups at 3 months and B vs. D represent comparison between groups at 6 months. The bolded values are the statistically significant values *p* < 0.05.

Abbreviations: DAS28-CRP, Disease Activity Score-28-C-reactive Protein; ACR20, American College of Rheumatology 20% improvement criteria; ACR50, American College of Rheumatology 50% improvement criteria; ACR70, American College of Rheumatology 70% improvement criteria; EULAR, european league against rheumatism response criteria; n, number; 95%CI, 95% Confidence Interval.

Significant clinical improvements in the digoxin group were observed in multiple disease measures in contrast to the control group concerning DAS28-CRP, DAS28-ESR, tender as well as swollen joint counts, the patient’s as well as the physician’s global assessment, pain assessment, morning stiffness, HAQ-DI score, CDAI, and SDAI after three and 6 months of treatment initiation (Table 3). Serum levels of CRP and ESR were also significantly decreased in the digoxin group in contrast to the control group at both time points (Table 4).

**TABLE 3:** Change in the clinical assessment outcomes after three and 6 months of treatment.

Characteristics	Control group (n = 30)			Digoxin group (n = 30)			*p*-value*			
	Baseline	3 months	6 months	Baseline	3 months	6 months	A vs. C	95%CI	B vs. D	95%CI
DAS28-CRP	5.4 ± 0.83	4.96 ± 0.99	4.46 ± 1.14	5.46 ± 0.78	4.34 ± 1	3.43 ± 1.31	**0.02** ^a^	0.1, 1.13	**0.002** ^a^	0.4, 1.67
DAS28-ESR	5.94 ± 0.88	5.43 ± 1.06	4.90 ± 1.19	6.01 ± 0.80	4.76 ± 1.06	3.72 ± 1.46	**0.017** ^a^	0.12, 1.22	**0.001** ^a^	0.49, 1.87
TJC	11 (7.8–13)	9 (5–12)	7 (3–10)	11 (8–13.3)	6.5 (4–9)	2.5 (1–7)	**0.043** ^b^	0.35, 4.65	**0.003** ^b^	1.05, 5.21
SJC	7 (5–9)	5.5 (3.75–8)	4.5 (2–6.3)	7 (5–9.3)	3.5 (2–5.3)	1 (0–3.3)	**0.022** ^b^	0.19, 3.27	**0.004** ^b^	0.68, 3.59
DAS28 class, n (%)										
Remission (≤2.6)	0	0	2 (6.7%)	0	1 (3.3%)	9 (30%)	**0.038** ^c^	2.5, 20.6	**0.028** ^c^	1.2, 17.8
Low (>2.6–3.2)	0	1 (3.3%)	4 (13.3%)	0	5 (16.7%)	8 (26.7%)				
Moderate (>3.2–5.1)	10 (33.3%)	15 (50%)	14 (46.7%)	11 (36.7%)	19 (63.3%)	8 (26.7%)				
High (>5.1)	20 (66.7%)	14 (46.7%)	10 (33.3%)	19 (63.3%)	5 (16.7%)	5 (16.7%)				
Patient’s Global Assessment of disease activity (0–100 mm, VAS)	53.5 ± 11.5	45.6 ± 13.6	42 (24.8–51.3)	53.9 ± 11.6	36.5 ± 15.4	26.5 (12.5–39.5)	**0.019** ^a^	1.53, 16.5	**0.007** ^b^	3.18, 19.5
Physician’s Global Assessment of Disease Activity (0–100 mm, VAS)	45.1 ± 11.3	38.2 ± 13.3	34.5 (18.8–45)	45.2 ± 11.5	29.9 ± 14.2	21.5 (8–31)	**0.022** ^a^	1.26, 15.5	**0.009** ^b^	2.5, 17.7
Patient’s assessment of pain (0–100 mm, VAS)	51.7 ± 11.6	44.1 ± 13.5	36.7 ± 15.1	51.9 ± 11.7	35.1 ± 15.3	25.6 ± 16.5	**0.019** ^a^	1.52, 16.4	**0.009** ^a^	2.86, 19.2
Morning stiffness (min)	65.7 ± 9.74	56.3 ± 10.3	46.2 ± 12.4	65.7 ± 9.6	49.3 ± 11	35.2 ± 13.4	**0.013** ^a^	1.56, 12.6	**0.002** ^a^	4.38, 17.7
HAQ-DI	1.77 (1.47–2.23)	1.67 (1.36–2.15)	1.5 (1.23–2)	1.87 (1.56–2.29)	1.45 (1.16–1.77)	1.18 (0.86–1.57)	**0.019** ^b^	0.08, 0.54	**0.003** ^b^	0.16, 0.64
CDAI	28.1 ± 9.56	23.3 ± 10.3	18.6 (9.5–26)	28.5 ± 9.12	17.3 ± 9	7.95 (3.6–17.2)	**0.02** ^a^	0.97, 11	**0.003** ^b^	2.45, 12.4
SDAI	30.6 ± 10.6	25.5 ± 11.4	20.6 (10.5–28.9)	31.2 ± 10.1	18.9 ± 9.9	8.7 (4–18.7)	**0.02** ^a^	1.05, 12.1	**0.003** ^b^	2.64, 13.7

Data are presented in terms of mean ± SD, median (inter-quartile range IQR), or number and percentages.

^a^ Unpaired student’s *t*-test.

^b^ Mann-Whitney U test.

^c^ Chi-square test.

*Between groups comparison where A vs. C represent comparison between groups at 3 months and B vs. D represent comparison between groups at 6 months. The bolded values are the statistically significant values *p* < 0.05.

Abbreviations: DAS28, Disease Activity Score-28; TJC, tender joint count; SJC, swollen joint count; HAQ DI, health assessment questionnaire disability index; CDAI, clinical disease activity index; SDAI, simplified disease activity index; 95%CI, 95% Confidence Interval.

**TABLE 4:** Change in the laboratory parameters after three and 6 months of treatment.

Parameters	Control group (n = 30)			Digoxin group (n = 30)			*p*-value*			
	Baseline	3 months	6 months	Baseline	3 months	6 months	A vs. C	95%CI	B vs. D	95%CI
IL-17A (pg/mL)	1.98 (1.73–2.59)	1.81 (1.59–2.44)	1.68 (1.28–2.32)	2.22 (1.91–2.64)	1.57 (1.25–1.96)	0.84 (0.61–1.39)	**0.01** ^b^	0.14, 0.78	**<0.001** ^b^	0.56, 1.2
IL-23 (pg/mL)	163 ± 21.2	145 ± 24.8	125 ± 29.1	165.8 ± 18.3	126 ± 19.2	86.9 ± 24.6	**0.001** ^a^	7.91, 3.85	**<0.001** ^a^	23.8, 51.7
HIF-1α (pg/mL)	26.2 ± 4.37	22.4 ± 4.64	17.9 ± 4.98	26.3 ± 3.28	17.9 ± 3.57	9.53 ± 3.71	**<0.001** ^a^	2.32, 6.6	**<0.001** ^a^	6.11, 10.7
VEGF (pg/mL)	276 ± 32.5	256 ± 34.4	232 ± 38	278 ± 27.9	231 ± 31.1	186.4 ± 32	**0.005** ^a^	7.51, 41.4	**<0.001** ^a^	27.7, 64
Th17 cells (%)	1.49 ± 0.37	1.28 ± 0.42	1.05 ± 0.46	1.53 ± 0.26	1.01 ± 0.26	0.41 ± 0.28	**0.003** ^a^	0.1, 0.46	**<0.001** ^a^	0.44, 0.84
CRP (mg/L)	24 (16.8–31.3)	21 (13.5–29.3)	17 (8.5–0.26)	24.5 (17.8–34.5)	14 (8.8–20.5)	8 (4–14)	**0.036** ^b^	0.44, 11.8	**0.009** ^b^	1.64, 13.2
ESR (mm/hr)	45 (32.8–53.0)	39.5 (24.5–48.5)	33.5 (20.8–43)	44 (40.5–54.3)	29.5 (20–37.5)	21 (11–31)	**0.018** ^b^	1.23, 15.4	**0.002** ^b^	3.8, 18.4
RF (IU/mL)	99.5 (43.3–128)	99.5 (41.8–125)	99 (40.3–127)	91.5 (64.8–127)	88 (62.5–113)	84.7 (57.3–106)	0.773^b^	−22.2, 28.4	0.668^b^	−20.7, 30
ACPAs (U/mL)	112.5 (11–232.5)	112.5 (10.8–232)	112.5 (10.8–232.5)	177.4 (17.8–225)	175.5 (18.5–223.3)	175 (18–222)	0.367^b^	−66.1, 63.3	0.311^b^	−65.8, 63.4
HB (g/dL)	11.3 ± 0.96	11.3 ± 0.96	11.3 ± 0.96	11.3 ± 1.19	11.3 ± 1.16	11.3 ± 1.14	0.898^a^	−0.52, 0.59	0.938^a^	−0.52, 0.57
WBCs (x10^3/µL)	8 ± 2.05	7.67 ± 2.02	7.33 ± 2.06	8.14 ± 1.72	6.62 ± 1.64	5.31 ± 1.65	**0.03** ^a^	0.1, 2	**<0.001** ^a^	1.07, 3
Platelets (x10^3/µL)	296 ± 55.9	285 ± 55.3	271 ± 55.6	301 ± 39.1	258 ± 36.3	240 ± 41.1	**0.03** ^a^	2.6, 51	**0.017** ^a^	5.8, 56.4
NLR	2.10 (1.38–2.48)	1.80 (1.20–2.33)	1.50 (0.98–2.13)	2.25 (1.98–2.7)	1.40 (1.1–1.55)	0.6 (0.5–0.9)	**0.028** ^b^	0.1, 1.3	**<0.001** ^b^	0.62, 1.62
PLR	136 ± 47	124 ± 39.3	112 ± 32.8	142.1 ± 24.6	105.1 ± 19.3	86.3 ± 17.7	**0.021** ^a^	2.9, 35	**<0.001** ^a^	11.7, 39
LMR	3.95 (2.93–5.05)	4.5 (3.38–6.2)	6.5 (4.35–7.65)	3.6 (2.88–3.93)	6 (5.23–7.03)	10.7 (8.9–13.5)	**0.002** ^b^	−2.28, −0.05	**<0.001** ^b^	−6.8, −3.3
SII	623 (362–855)	521 (301.8–690)	381 (237–623)	661 (549–896)	347 (271–433)	141 (118.5–219)	**0.013** ^b^	65.1, 446	**<0.001** ^b^	188, 491
SIRI	1.15 (0.7–1.71)	0.91 (0.53–1.39)	0.55 (0.36–1.04)	1.34 (1.17–1.91)	0.58 (0.44–0.7)	0.18 (0.13–0.23)	**0.004** ^b^	0.23, 1.15	**<0.001** ^b^	0.39, 0.97

Data are presented in terms of mean ± SD, or median (inter-quartile range IQR).

^a^ Unpaired Student’s *t*-test.

^b^ Mann-Whitney U test.

*Between groups comparison where A vs. C represent comparison between groups at 3 months and B vs. D represent comparison between groups at 6 months. The bolded values are the statistically significant values *p* < 0.05.

Abbreviations: IL, interleukin; HIF-1α, Hypoxia-inducible factor-1α; VEGF, vascular endothelial growth factor; Th17 cells, T-helper 17 cells; ESR, erythrocyte sedimentation rate; CRP, C-reactive Protein; RF, rheumatoid factor; ACPAs, anti-citrullinated peptide antibodies; HB, hemoglobin; WBCs, White blood cells; NLR, neutrophil to lymphocyte ratio; PLR, platelet to lymphocyte ratio; LMR, lymphocyte to monocyte ratio; SII, systemic immune inflammation index; SIRI, systemic inflammation response index; 95%CI, 95% Confidence Interval.

Additionally, the inflammatory markers derived from hematological analysis or peripheral blood cell count ratio of RA patient samples, such as NLR, PLR, SII, and SIRI, which reflect systemic inflammation, significantly decreased after three and 6 months of treatment in the digoxin group in contrast to the control group (*p* = 0.028, *p* < 0.001; *p* = 0.021, *p* < 0.001; *p* = 0.013, *p* < 0.001; *p* = 0.004, *p* < 0.001, respectively). However, a significant elevation in the LMR in the digoxin group relative to the control group was observed at both measured time points (*p* = 0.002 and *p* < 0.001, respectively).

### 3.3 Serum levels of biological markers at baseline and after treatment

Baseline serum concentrations of the inflammatory markers IL-17A and IL-23 and the angiogenic factors HIF-1α and VEGF were similar at baseline in all study groups (*p* > 0.05). However, digoxin treatment led to a substantial decline in serum concentrations of these biomarkers following three and 6 months of treatment in contrast to the control group (*p* < 0.05) (Table 4).

### 3.4 Th17 cell proportions in PBMCs at baseline and after treatment

In the digoxin group, analysis of peripheral blood elucidated significantly diminished proportions of Th17 cells at both measured time points in contrast to the control group (*p* = 0.003 and *p* < 0.001, respectively) (Table 4).

### 3.5 Correlation analysis

Pearson’s correlation analysis was employed for both groups to uncover the potential relationships between inflammatory biomarkers and clinical outcomes as well as laboratory variables. The study identified strong statistically significant positive correlations between Th17 cell proportions and serum concentrations of IL-17A, IL-23, HIF-1α, and VEGF (r = 0.843, *p* < 0.001; r = 0.817, *p* < 0.001; r = 0.850, *p* < 0.001; r = 0.759, *p* < 0.001, respectively). Similarly, Th17 cells, as well as serum concentrations of IL-17A, IL-23, HIF-1α, and VEGF, exhibited significant positive correlations with DAS28-CRP, DAS28-ESR, TJC, SJC, CRP, ESR, PtGA, PhGA, CDAI, SDAI, HAQ-DI, NLR, PLR, SII, and SIRI, and a significant negative correlation with LMR (Table 5).

**TABLE 5:** Correlations between inflammatory markers and clinical indicators.

Variables	Serum IL-17A	Serum IL-23	Th17 cells (%)	Serum HIF-1α	Serum VEGF
	(*r*)	(*r*)	(*r*)	(*r*)	(*r*)
DAS28-CRP	0.78	0.71	0.76	0.74	0.76
DAS28-ESR	0.78	0.71	0.75	0.75	0.77
TJC	0.79	0.70	0.76	0.74	0.73
SJC	0.74	0.65	0.72	0.70	0.68
CRP	0.76	0.71	0.75	0.72	0.74
ESR	0.80	0.71	0.78	0.76	0.78
PtGA	0.74	0.66	0.72	0.72	0.72
PhGA	0.73	0.67	0.71	0.71	0.71
CDAI	0.78	0.69	0.75	0.74	0.73
SDAI	0.78	0.69	0.75	0.74	0.73
HAQ-DI	0.86	0.77	0.76	0.77	0.80
NLR	0.77	0.67	0.82	0.78	0.64
PLR	0.51	0.50	0.54	0.46	0.40
LMR	−0.57	−0.51	−0.53	−0.57	−0.43
SII	0.85	0.76	0.86	0.83	0.73
SIRI	0.83	0.76	0.82	0.84	0.72

For all correlations *p* < 0.01.

### 3.6 Association of baseline demographic characteristics with clinical outcomes

To increase the robustness of the results, multivariate logistic and linear regression analyses were carried out to investigate the impact of baseline demographic factors and digoxin treatment on the clinical outcomes assessed for RA patients. Results from multivariate logistic regression (Table 6) for ACR20, ACR50, and ACR70 response rates as dependent variables, and multivariate linear regression (Table 7) for DAS28-CRP, CDAI, SDAI, and morning stiffness as dependent variables, demonstrated no significant associations between demographic factors and clinical outcomes in RA patients. While for digoxin treatment, there was a significant association between patients receiving digoxin and the clinical responses achieved according to the ACR20, ACR50, and ACR70 criteria, indicating that patients receiving digoxin are more likely to achieve clinical improvement compared to those not receiving it (Table 6). Additionally, digoxin treatment demonstrated significant associations with the clinical outcomes including DAS28-CRP, CDAI, SDAI, and morning stiffness, suggesting that it is associated with a reduction in disease activity and symptom severity, indicating its potential beneficial role in RA patients (Table 7).

**TABLE 6:** Multivariate binary logistic regression analysis of independent predictors for clinical response in rheumatoid arthritis patients.

Variables	ACR20		ACR50		ACR70	
Factors	OR (95% Cl)	*p*-value	OR (95% Cl)	*p*-value	OR (95% Cl)	*p*-value
Age (years)	0.963 (0.868,1.07)	0.483	1.04 (0.932, 1.16)	0.492	0.909 (0.778, 1.06)	0.228
Gender	0.522 (0.11, 2.47)	0.412	0.851 (0.421, 1.556)	0.465	0.264 (0.045, 1.55)	0.140
BMI (kg/m^2)	2.23 (0.79, 6.32)	0.131	0.593 (0.195, 1.81)	0.358	0.195 (0.034, 1.12)	0.067
Disease Duration	1.28 (0.97, 1.69)	0.084	1.11 (0.830, 1.45)	0.519	1.49 (0.957, 2.32)	0.078
Digoxin Treatment	4.2 (1.15, 15.3)	**0.03**	4.16 (1.18, 14.7)	**0.027**	11.1 (1.36, 88.6)	**0.024**

OR (95% CI) odds ratios (95% confidence intervals); ACR, american college of rheumatology improvement criteria; BMI, body mass index. The bolded values indicate statistically significant data (*p* < 0.05).

**TABLE 7:** Multivariate linear regression analysis of independent predictors for clinical outcomes in rheumatoid arthritis patients.

Variables	DAS28-CRP		CDAI		SDAI		Morning stiffness	
Factors	B (95% Cl)	*p*-value	B (95% Cl)	*p*-value	B (95% Cl)	*p*-value	B (95% Cl)	*p*-value
Age (years)	0.025 (−.029, .078)	0.355	0.20 (−.215, .623)	0.333	0.221 (−.24, .69)	0.345	0.23 (−.317, .776)	0.404
Gender	1.82 (.029, 3.62)	0.047	11.7 (−2.35, 25.8)	0.101	12.9 (−2.7, 28.5)	0.104	3.88 (−1.51, 22.2)	0.121
Smoking	−0.976 (−2.88, .928)	0.308	−5.01 (−19.9, 9.9)	0.504	−5.45 (−22.1, 11.2)	0.513	−14.7 (−34.2, 4.76)	0.136
BMI (kg/m^2)	−0.062 (−.586, .461)	0.812	−0.93 (−5.04, 3.17)	0.650	−1.07 (−5.63, 3.49)	0.640	0.105 (−5.25, 5.46)	0.969
Disease Duration	−0.075 (−.214, .063)	0.280	−0.73 (−1.82, .352)	0.181	−.806 (−2.01, .402)	0.186	−0.290 (−1.71, 1.13)	0.683
Digoxin Treatment	−0.937 (−1.57, −.305)	**0.004**	−6.8 (−11.8, −1.84)	**0.008**	−7.47 (−13, −1.96)	**0.009**	−9.79 (−16.3, −3.32)	**0.004**

DAS28-CRP, Disease Activity Score-28-C-reactive Protein; CDAI, clinical disease activity index; SDAI, simplified disease activity index; 95% CI (95% confidence intervals). The bolded values indicate statistically significant data (*p* < 0.05).

### 3.7 Clinical adverse effects

The absence of statistically significant differences in the adverse effects encountered by the digoxin and control groups is evident from the data presented in Table 8.

**TABLE 8:** Therapy-related adverse effects were reported as number per group after 3 and 6 months.

Adverse effects	Control group (n = 30)		Digoxin group (n = 30)		*p*-value	
	3 months	6 months	3 months	6 months	3 months (control vs. digoxin)	6 months (control vs. digoxin)
Malaise	1 (3.3%)	2 (6.7%)	2 (6.7%)	4 (13.3%)	0.554	0.389
Dizziness	1 (3.3%)	2 (6.7%)	2 (6.7%)	3 (10%)	0.554	0.640
Blurred vision	0%	1 (3.3%)	0%	2 (6.7%)	—	0.554
Nausea	2 (6.7%)	3 (10%)	1 (3.3%)	2 (6.7%)	0.554	0.640
Vomiting	0%	1 (3.3%)	0%	1 (3.3%)	1.00	1.00
Diarrhea	1 (3.3%)	2 (6.7%)	1 (3.3%)	1 (3.3%)	1.00	0.554
Anorexia	2 (6.7%)	3 (10%)	1 (3.3%)	2 (6.7%)	0.554	0.640
Abdominal pain	0%	1 (3.3%)	1 (3.3%)	2 (6.7%)	0.313	0.554
Dry mouth	1 (3.3%)	1 (3.3%)	2 (6.7%)	3 (10%)	0.554	0.301
Fatigue	0%	2 (6.7%)	1 (3.3%)	3 (10%)	0.313	0.640
Confusion	1 (3.3%)	1 (3.3%)	1 (3.3%)	2 (6.7%)	1.00	0.554
Weakness	1 (3.3%)	2 (6.7%)	2 (6.7%)	3 (10%)	0.554	0.640
Hair loss	1 (3.3%)	3 (10%)	2 (6.7%)	4 (13.3%)	0.554	0.688
Headache	1 (3.3%)	3 (10%)	3 (10%)	5 (16.7%)	0.301	0.448
Insomnia	0%	2 (6.7%)	1 (3.3%)	3 (10%)	0.313	0.640
Anxiety	0%	1 (3.3%)	0%	1 (3.3%)	—	1.000
Rash	0%	1 (3.3%)	0%	2 (6.7%)	—	0.554
WBC count decreased	1 (3.3%)	3 (10%)	1 (3.3%)	4 (13.3%)	1.00	0.688
Pharyngitis	1 (3.3%)	3 (10%)	1 (3.3%)	4 (13.3%)	1.00	0.688
Hypokalemia	0%	2 (6.7%)	0%	0%	—	0.150
Arrhythmia	0%	0%	0%	0%	—	—
ALT increased	2 (6.7%)	5 (16.7%)	0%	2 (6.7%)	0.150	0.228
AST increased	2 (6.7%)	5 (16.7%)	0%	2 (6.7%)	0.150	0.228

Arrhythmia and other cardiac manifestations were not observed in any of the patients in either 302 group. No toxicities attributable to digoxin were observed, and the other adverse effects experienced 303 by the patients in both groups were mild, tolerable, temporary, and resolved spontaneously without 304 needing any specific intervention or treatment discontinuation. Routine evaluation of ECG, CBC, serum electrolytes (Na+, K+, total Ca+2, ionized Ca+2 305 ), and kidney as well as liver function tests 306 confirmed that neither digoxin nor csDMARDs revealed any serious adverse events in either of the 307 two groups.

## 4 Discussion

This randomized, prospective, double-blinded, and placebo-controlled clinical study is the first to evaluate the potential therapeutic effect of digoxin as an adjunctive therapy to csDMARDs on RA disease activity. Beyond their recognized therapeutic effect in cardiac diseases, cardiac glycosides are gaining significant attention for their established anti-inflammatory properties and the interesting possibility of immunomodulatory effects. Recent research suggests that digoxin has a promising therapeutic potential in treating non-cardiac diseases, such as gastrointestinal diseases, including steatohepatitis, as well as viral infections, cancer, and other metabolic disorders. Intriguingly, studies have shown that small doses of digoxin are safe and can exert effective biological activity as they selectively inhibit Th17 cell differentiation (Jamshed et al., 2023).

In the present study, we evaluated the effect of digoxin on Th17 cell proportions in PBMCs of RA patients, as well as other inflammatory and angiogenic markers involved in RA’s pathogenesis and their association with treatment response and improved disease activity.

We found a significant decline in the DAS28-CRP and CDAI with an average of −2.03 and −17.20 in the digoxin group compared to a decline of −0.94 and −9.41 in the control group following 6 months of treatment. Furthermore, active RA patients in the digoxin group exhibited a significantly improved response based on ACR and EULAR response criteria and improved patients’ QOL, offering a potential advantage over patients who received csDMARDs alone.

The observed clinical improvement in the digoxin group could be an outcome of its anti-inflammatory properties, as it suppresses the key cytokine mediators of the inflammatory response involving IL-1β, IL-6, IL-17A, and IL-23; it also inhibits IL-23-induced Th17 cell differentiation in murine models (Huh et al., 2011; Saeed et al., 2020). Our study also revealed the down-regulating effect of digoxin on the inflammatory cytokine mediators IL-17A and IL-23, which, in turn, can effectively suppress Th17 cell proliferation.

It has been demonstrated that the high percentages of Th17 cells were correlated with elevated DAS28 scores and CRP levels (Schinocca et al., 2021; Zizzo et al., 2011). In the present study, we found that flow cytometric analysis of Th17 cells in the digoxin group showed a substantial decrease in the Th17 cell proportions in contrast to the control group.

In agreement with our results, Saeed et al. have found that digoxin treatment suppresses differentiation of the Th17 cell in the cultured PBMCs treated with digoxin and obtained from RA patients whose sample size was comparable to ours (Saeed et al., 2020). Also, Zaczkiewicz et al. have shown that digoxin treatment could significantly reduce CRP plasma levels in decompensated heart failure patients (Zaczkiewicz et al., 2022). The observed improvement of CRP within the digoxin group likely arises from its inherent anti-inflammatory activity, as it reduced the levels of IL-1β and IL-6 since these cytokine mediators are known to stimulate CRP synthesis in hepatocytes through NF-κB and STAT3 signaling pathways (Kleemann et al., 2003). Despite our sample size, the findings of this study are consistent with those reported in previous investigations with comparable participant numbers that provide evidence suggesting a potential benefit of digoxin for RA. However, larger-scale randomized controlled trials are warranted to confirm these observations and to assess the long-term efficacy and safety profile of the digoxin.

Several data indicate that the inflammatory cytokine mediator IL-23 is included in Th17 cell activation and differentiation with subsequent IL-17A production that actively participates in tissue inflammation and destruction, confirming its pivotal role in RA’s pathogenesis (Schinocca et al., 2021; Tsukazaki and Kaito, 2020). In light of these data, it has been shown that digoxin’s immunomodulatory and anti-inflammatory outcomes in RA patients are potentially mediated by downregulating Th17 cell populations with a subsequent reduction of serum IL-23 and IL-17A levels.

Therefore, augmenting csDMARD therapy with digoxin in RA patients can effectively downregulate Th17 cell populations, which is pivotal in RA’s inflammatory cascade and disease progression. This effect can be explained by digoxin’s potent inhibition of the transcriptional activity of RORγt, the master regulator of Th17 cell development (Ma et al., 2017; Saeed et al., 2020; Zhenjiang Hou and Wang, 2019).

Our results are consistent with the observations of Huh et al. who exhibited that digoxin suppresses Th17 differentiation in murine models by inhibiting the transcriptional activity of RORγt (Huh et al., 2011). In addition, Fujita-Sato et al. have demonstrated that digoxin is a RORγt antagonist with promising therapeutic benefits against Th17-driven autoimmune diseases (Fujita-Sato et al., 2011). Also, it was found that digoxin treatment could exert an anti-inflammatory effect and reduce the severity of autoimmune encephalomyelitis (EAE) and Crohn’s disease in an experimental model by selectively reducing the proportion of Th17 cells and through the downregulation of mRNAs associated with Th17-related cytokines (Huh et al., 2011; Tani et al., 2017).

Several studies have demonstrated that HIF-1α regulates Th17 signature genes by directly activating the transcription of RORγt, which promotes Th17 development (Dang et al., 2011; Paradowska-Gorycka et al., 2020). Additionally, research has demonstrated that digoxin inhibits inflammasome activity maintained by the HIF-1α pathway in a nonalcoholic steatohepatitis (NASH) mouse model, thereby validating its role as an inhibitor of HIF-1α activation (Ouyang et al., 2018; Zhang et al., 2008; Zhao et al., 2019). Also, it was noted that digoxin inhibited the growth of prostate cancer cells in a murine model by downregulating HIF-1α-dependent gene expression and suppressing its protein synthesis (Platz et al., 2011). Furthermore, Wei et al. showed that digoxin inhibited non-small-cell lung cancer cell growth as it acts as a potent inhibitor of HIF-1α synthesis in A549 cells, causing downregulation of hypoxia-induced VEGF and NDRG1 overexpression (Wei et al., 2013). While this is an area of ongoing research and there is currently a lack of clinical studies specifically assessing the antiangiogenic effects of digoxin. This study is the first to evaluate the potential anti-angiogenic effects of digoxin in RA patients. Our results may be consistent with the previous experimental studies, which revealed that digoxin may possess anti-angiogenic properties through suppression of the angiogenic markers HIF-1α and VEGF, suggesting that digoxin may have potential antiangiogenic properties in RA patients and exert its therapeutic effects through HIF-1α/VEGF axis suppression.

We observed that the NLR, PLR, and the novel immune-inflammatory markers SII and SIRI were significantly reduced, and LMR was markedly elevated in the digoxin group in contrast to the control group after three and 6 months of treatment, which confirmed digoxin’s anti-inflammatory outcomes in RA patients. In addition, positive relationships were observed between these markers and both laboratory and clinical indicators of disease activity in the studied groups, indicating their prognostic value in assessing RA disease activity.

Zhou et al. (2023) showed that the elevation of NLR contributed independently to higher risks of cardiovascular mortality in RA patients. Furthermore, studies revealed that high neutrophil and monocyte levels, along with reduced lymphocytes, are independent risk factors for cardiovascular disease (Çırakoğlu Ö and Yılmaz, 2021; Targonska-Stepniak and Grzechnik, 2023; Walzik et al., 2021). Therefore, we can expect that digoxin may have protective effects against cardiovascular risks among RA patients, as it inhibits NLR, which is considered an independent predictor of cardiovascular risks among those patients, so further future clinical studies regarding the protective effects of digoxin for cardiovascular risk prevention are required in RA patients.

Baseline demographic characteristics, including age, sex, body mass index (BMI) and disease duration, were not significant predictors of clinical response to various treatments in RA as assessed by both logistic and linear regression models. These findings suggest that these factors did not influence the treatment outcomes in our study population. In consistent with our results (Jassim and Redha, 2021; Mirpourian et al., 2014; Narvaez et al., 2011; Pers et al., 2015) have indicated that demographic factors do not significantly influence treatment outcomes in rheumatoid arthritis patients. However, patients receiving digoxin, when adjusted for other variables such as age, sex, BMI, and disease duration, experienced significant improvements in various clinical measures and increased the likelihood of achieving clinically meaningful improvements in response criteria, suggesting that digoxin may play a beneficial role in the management of RA.

Overall, no major safety concerns arising from digoxin use in RA patients were identified during the study period. Furthermore, no clinically relevant pharmacokinetic interactions between digoxin and csDMARDs were reported.

Despite the encouraging results regarding the usage of digoxin as an adjuvant therapy in managing RA, our trial had certain limitations: the relatively small sample size and the recruitment of RA patients from a single medical center; therefore, we cannot fully generalize our findings until further research is conducted at multiple centers. Furthermore, we did not perform a radiological evaluation to ascertain the extent of radiological changes during treatment and correlate this with laboratory and clinical improvement; however, significant radiographic changes may not have been detected due to the brief study period. Therefore, we can describe the contribution of digoxin in treating RA as an adjuvant or complementary therapy, and we still need to learn more about its effectiveness through comprehensive, large-scale, multi-center clinical trials carried out for extended follow-up periods to provide deeper insights into digoxin’s therapeutic action in RA.

## 5 Conclusion

In conclusion, our results suggest that the co-administration of digoxin can improve and strengthen the effect of conventional treatment in RA patients, as indicated by better patient response and lower disease activity, supporting previous studies implying that drugs with anti-inflammatory properties might exhibit an enhanced anti-rheumatic impact.

## Data Availability

The original contributions presented in the study are included in the article/supplementary material, further inquiries can be directed to the corresponding author.
